# Clinical efficacy and influencing factors of percutaneous kyphoplasty for osteoporotic vertebral compression fractures: a 10-year follow-up study

**DOI:** 10.1186/s12893-024-02322-5

**Published:** 2024-01-19

**Authors:** Zihao Zhan, Ran Li, Dongming Fu, Hao Han, Yiang Wu, Bin Meng

**Affiliations:** Department of Orthopaedics, The First Affiliated Hospital of Soochow University, Suzhou, 215006 Jiangsu China No.899 Pinghai Road,

**Keywords:** Osteoporotic fractures, Kyphoplasty, Patient outcome assessment, Risk factors

## Abstract

**Background:**

To date, few reports have evaluated the long-term outcome of percutaneous kyphoplasty (PKP) for osteoporotic vertebral compression fractures (OVCFs) and the factors influencing the long-term outcome of this procedure are uncertain.

**Methods:**

A total of 91 patients underwent PKP for thoracolumbar OVCFs from June 2012 to December 2012. Pain Visual Analogue Scores (VAS) and Oswestry Disability Index (ODI) were recorded preoperatively and after 10-year follow-up. Factors that may affect surgical outcome, such as gender, age, height, weight, hypertension, diabetes, cause of injury, fracture segment, length of hospitalization, history of previous spinal surgery, preoperative bone mineral density (BMD), preoperative VAS and ODI scores, length of surgery, bone cement dosage, postoperative standardized anti-osteoporosis treatment, and other new vertebral fractures, were analyzed by multiple linear regression with VAS and ODI scores at the last follow-up. The correlation factors affecting the efficacy were analyzed.

**Results:**

The preoperative and final follow-up pain VAS was 7.9 ± 1.1 and 2.2 ± 1.1. ODI scores were 30.4 ± 4.2 and 10.7 ± 2.6. The difference was statistically significant (*P* < 0.05). Most of the patients were females aged 65–75 years who suffered low-energy injuries, with most of the fracture segments in the thoracolumbar region (T11-L2). At the final follow-up visit, 12 cases (13.19%) developed other new vertebral fractures, and 33 cases (36.26%) continued to adhere to anti-osteoporosis treatment after discharge. Multiple linear regression analysis showed that there was a statistical difference between gender and VAS score at the last follow-up (*P* < 0.05), and between age, cause of injury and postoperative standardized anti-osteoporosis treatment and ODI at the last follow-up (*P* < 0.05). There were no statistically significant differences between the other factors and the final follow-up VAS and ODI scores (*P* > 0.05).

**Conclusion:**

The long-term outcome after PKP is satisfactory. Age, gender, cause of injury, and standardized postoperative anti-osteoporosis treatment may be factors affecting the long-term outcome.

**Supplementary Information:**

The online version contains supplementary material available at 10.1186/s12893-024-02322-5.

## Introduction

Osteoporosis (OP) is an abnormality in bone metabolism and destruction of bone microarchitecture due to multiple causes, leading to increased bone fragility and fracture risk [1]. Osteoporotic vertebral compression fractures (OVCFs) are spinal fractures that occur under minor trauma due to osteoporosis-induced bone loss [2]. The main clinical manifestations include widespread thoracolumbar back pain that lasts for weeks to months, mobility dysfunction, and kyphotic deformity, which seriously affects patients' quality of life. Traditional treatments for OVCFs include bed rest, narcotic analgesic applications, braces, and physical therapy. However, these traditional therapies negatively affect both muscle strength and bone mass and can lead to serious complications. Percutaneous kyphoplasty (PKP) is the current minimally invasive surgical treatment commonly used to treat OVCFs [3]. This technique uses percutaneous puncture and balloon expansion to reposition the collapsed vertebral body and stabilize the diseased vertebral body by bone cement infusion to reduce the pain associated with the fracture [4]. It has the advantages of short surgical time, little trauma, and early postoperative bed mobility [5], and provides rapid relief of back pain in a short period of time. Most studies claim that the short-term outcome of this procedure is satisfactory [6, 7] and patients report significant pain relief, independence in getting out of bed, and improved quality of life after surgery. However, there are few studies on its long-term efficacy. The purpose of this study is to evaluate the long-term efficacy of PKP surgery, to analyze the factors that may affect the long-term efficacy of the surgery, to determine whether the long-term outcome of the surgery is satisfactory, and to guide clinical treatment by understanding the factors that affect the outcome of the surgery.

## Patients and methods

### Patients

From June 2012 to December 2012, a total of 129 OVCF patients who underwent thoracolumbar PKP were selected from the First Affiliated Hospital of Soochow University according to the inclusion and exclusion criteria. Finally, 100 patients were selected for follow-up using randomized sampling, and 9 patients were lost at the final follow-up; a total of 91 patients were included, including 24 males and 67 females, aged 55–86 years.

### Selection criteria

Inclusion criteria: (1) preoperative X-ray, CT and MRI examination clearly showed that single-segment vertebral compression fracture occurred in the spine, and MRI examination showed intravertebral edema, low signal in T1-weighted image and high signal change in T2-weighted image, and preoperative patients all had different degrees of low back pain and local spinous percussion pain; (2) preoperative bone density T value ≤ -2.5 SD; (3) complete clinical data.

Exclusion criteria: (1) pathological fractures caused by malignant tumors (such as breast cancer, prostate cancer, etc.); (2) combined spinal cord injury and neurological symptoms; (3) combined serious neurological and cardiopulmonary diseases and other contraindications to surgery.

### General information

According to the inclusion and exclusion criteria, a total of 91 patients were treated, including 24 males and 67 females. From January 2012 to December 2012, the clinical data of patients with osteoporotic vertebral compression fractures were retrospectively analyzed. The patients were followed up for 10 years. Pain Visual Analogue Scores (VAS) and Oswestry Disability Index (ODI) before and 10 years after surgery were recorded, such as gender, age, height, weight, hypertension, diabetes, cause of injury, fracture segment, length of hospitalization, history of previous spinal surgery, preoperative bone mineral density (BMD), preoperative VAS and ODI scores, length of surgery, bone cement dosage, postoperative standardized anti-osteoporosis treatment, and other new vertebral fractures.

### Surgical procedure

The procedure was standardized and the procedure was the same for all patients. After general anesthesia, the patient was placed in the prone position, routinely disinfected and towel dried, the surgical area exposed and skin protective film applied. After the fluoroscopy of the injured vertebra was correct, the needle was first taken into the 1.5 cm near the 10 o 'clock direction of the injured vertebra, and the needle was cut longitudinally for about 1 cm with a sharp knife. Then the puncture trocar passed through the skin, subcutaneous tissue, fascia and paraspinal muscle to the pedicle in turn. When the needle tip in the C-arm machine fluoroscopic position was located in the center of the vertebral arch, and the needle tip was located in the center of the lateral vertebral arch, the tapping of the needle was continued. When the tip of the needle is positioned in the orthogonal view of the internal marginal arch and the tip of the needle has entered the posterior vertebral body edge in the lateral view and the needle continues to enter about 0.5 cm, then the core of the needle is retracted and the working cannula, and the working channel edge probes before reaching the anterior edge of the injured vertebral body and does not penetrate into the bone. After a 2 cm thin hole is drilled, a balloon dilator is placed and an appropriate amount of contrast material is injected. Similarly, the contralateral side of the injured vertebral arch was puncturing simultaneously, and after cannula placement and balloon dilation, bone cement was prepared. After the cement reached the early mass phase, the puncture needle core was removed and the cement was slowly pushed in from both sides, stopping when the cement approached the posterior wall of the vertebral body. After the wound healed completely, the tube was extubated and sutured. 1–2 days after the operation, patients are recommended to wear a waist girdle to get out of bed and perform early rehabilitation training, in addition to routinely giving calcium and vitamin D anti-osteoporosis treatment.

### Observation index and follow-up

Electronic case data were collected from patients, and gender, age, height, weight, hypertension, diabetes mellitus, cause of injury, fracture segment, length of hospitalization, history of previous spine surgery, preoperative BMD, length of surgery, bone cement dosage, postoperative standardized anti-osteoporosis treatment, and other new vertebral fractures were recorded. The VAS score and ODI score were used to evaluate the preoperative status and postoperative efficacy of the patients. The preoperative BMD T-value was the mean BMD T-value from L1 to L5 measured by our dual-energy X-ray bone densitometry. Postoperative standardized anti-osteoporosis treatment: long-term oral calcium and osteopontin after discharge, and regular use of one of zoledronic acid, disulfiramab or bisphosphonates on this basis (use bisphosphonates with caution in patients with renal dysfunction and zoledronic acid with caution in patients with low blood calcium). Final follow-up included telephone follow-up, outpatient follow-up, and admission of patients with vertebral re-fractures. Telephone follow-up was performed using a uniform questionnaire that included current VAS and ODI scores, postoperative functional recovery, receipt of standardized anti-osteoporotic therapy after discharge from the hospital, and incidence of vertebral re-fracture, and patients were advised to undergo outpatient follow-up. Outpatient follow-up controls were consistent and included a physical examination, frontal and lateral radiographs of the thoracolumbar spine, and an MRI if the patient had recently developed pain symptoms.

### Statistical methods

SPSS 26. 0 statistical software (SPSS Inc.Chicago, IL) was used. Measurements were tested for normality, were normally distributed, and are expressed as mean ± standard deviation (x ® ± s), and paired T test analysis was performed for VAS and ODI scores before surgery, 1 day after surgery, 1 year after surgery, and at the last follow-up, and *P* < 0.05 was considered statistically different. Multiple linear regression stepwise selection method was used to correlate factors that may affect the outcome, such as gender, age, height, weight, hypertension, diabetes mellitus, cause of injury, fracture segment, length of hospitalization, history of previous spine surgery, preoperative BMD, preoperative VAS and ODI scores, length of surgery, bone cement dosage, postoperative standardized anti-osteoporosis treatment, and other new vertebral fractures, with the final follow-up VAS and ODI scores, respectively. scores for correlation analysis. All tests were two-sided. *P* < 0.05 was considered a statistically significant difference.

## Results

### General information

**Fig. 1:**
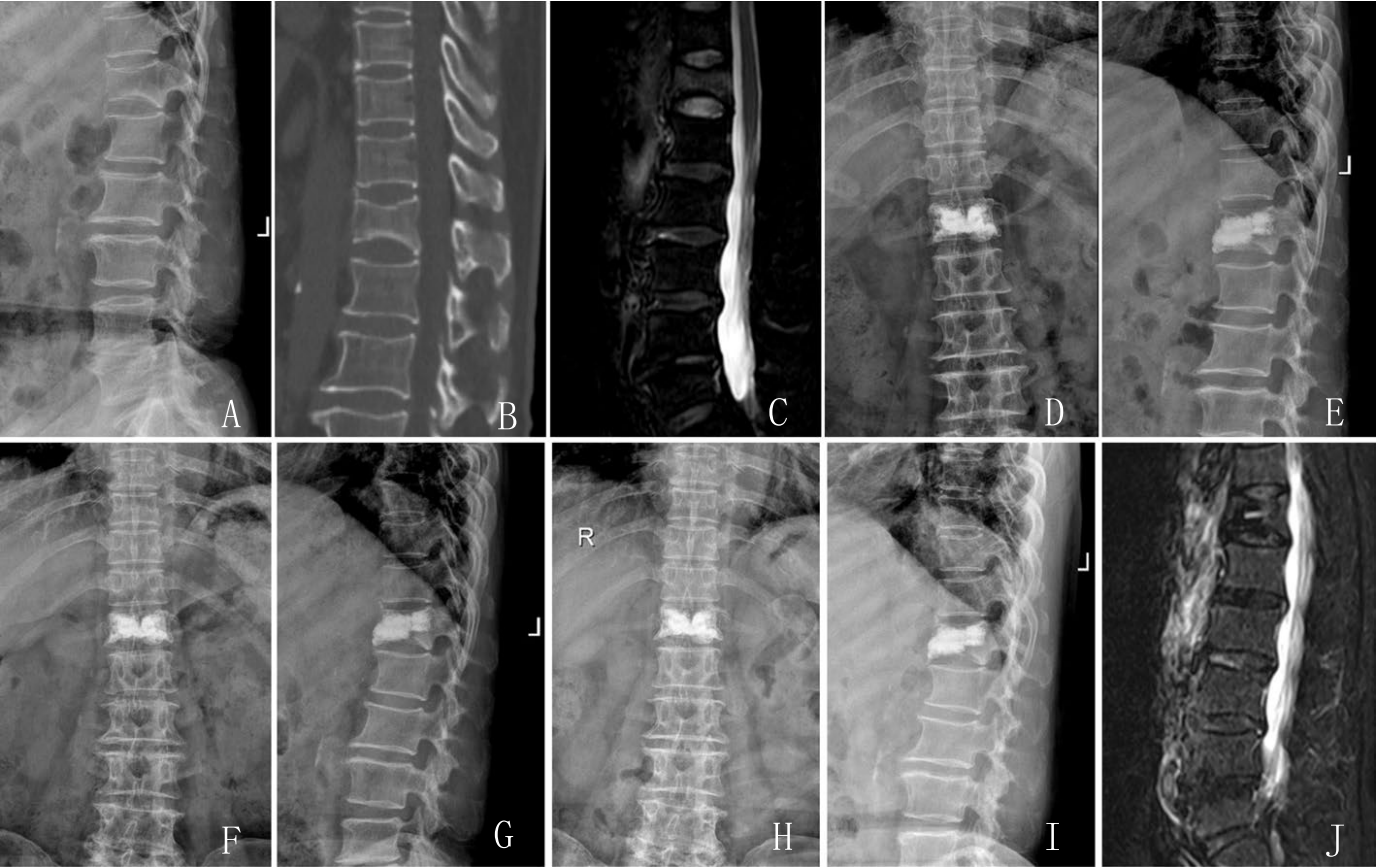
A 64-year-old female patient with OVCFs underwent PKP in June 2012. **A**-**C** Preoperative X-ray, CT, MRI showed fresh compression fracture of T12 vertebrae. **D** and **E** One day postoperative frontal and lateral spine radiographs showed that the cement was in place and well distributed in the T12 vertebral body. **F** and **G** Patients come to the clinic for review one month after surgery. **H** and **I** Patients come to the clinic for review one year after surgery. **J** At the last follow-up visit, MRI showed cement in place and no vertebral re-fracture

All 91 patients were followed up for 10 years, of which 67 were female (73.63%) and 24 were male (26.37%), aged 55–86 years (68.1 ± 8.0 years). The cause of first admission for spinal fracture was low energy injury, out of which 50 (54.95%) were inadvertent fall while walking on level ground, 8 (8.79%) were minor traffic accidents, 7 (7.69%) were fall from a low place, and 26 (28.57%) were sudden onset of low back pain without any apparent cause including while turning over or bending over in bed. The fractured segments were located in the thoracic segment (T10 and above) in 8 cases (8.79%), in the thoracolumbar segment (T11-L2) in 74 cases (81.32%), and in the lumbar segment (L3 and below) in 9 cases (9.89%). 48 patients were followed up by telephone and 31 patients came to the outpatient clinic for follow-up, all of whom underwent thoracolumbar spine X-ray and 6 had thoracolumbar spine MRI (Fig. 1). During the follow-up period, a total of 12 patients were admitted for vertebral re-fracture, with 7 (58.33%) re-fractures occurring adjacent to the operated vertebral body and 5 (41.67%) located in non-adjacent areas. 70 patients reported significant relief of low back pain on the first to third postoperative day and no significant low back pain in the past 10 years, giving positive feedback on the efficacy of surgery. Nine patients reported relief of pain symptoms during hospitalization, but mild pain in the low back still occurred after discharge when there was a change in weather or after prolonged activity, but it did not affect their life.

### Clinical outcomes

The VAS score was 2.2 ± 1.1 and ODI score was 10.7 ± 2.6 at the final follow-up, which were significantly improved compared with the preoperative scores, and the difference was statistically significant (*P* < 0.05) (Table 1). Some of the factors that may affect the long-term outcome after PKP and the assigned results are shown below (Table 2). The results of multiple linear regression analysis (Table 3) showed that there was a significant difference between VAS score at the last follow-up and gender (*P* < 0.05), and female patients had higher VAS score at the last follow-up than males; there was a statistical difference between ODI score at the last follow-up and age, cause of injury and standardized postoperative anti-osteoporosis treatment (*P* < 0.05). Postoperative ODI scores were higher in patients with older age, greater injury-causing violence, and no standardized postoperative anti-osteoporosis treatment. There was no significant difference between final VAS and ODI scores and height, weight, hypertension, diabetes mellitus, fracture segment, length of hospitalization, history of previous spine surgery, preoperative BMD, preoperative VAS and ODI scores, length of surgery, bone cement dosage, and other new vertebral fractures (*P* > 0.05).

**Table 1:** VAS and ODI scores before surgery and during follow-up

	PRE	1 DAY	1 YEAR	FINAL
VAS score	7.9 ± 1.1	3.6 ± 1.2*	2.4 ± 1.4*	2.2 ± 1.1*
ODI score	30.4 ± 4.2	24.3 ± 4.4*	13.6 ± 3.1*	10.7 ± 2.6*

*VAS* Visual Analogue Scale, *ODI* Oswestry Disability Index, *PRE* Preoperative, *1 DAY* One day after surgery, *1 YEAR* One month after surgery, *FINAL* Final follow-up

^*^ Significance compared with the preoperative, *P* < 0.05

**Table 2:** Some of the factors that may affect long-term efficacy and their assignments

Variables	Influencing Factor	Assignment	Result
X1	Gender	Male(1), Female(0)	1 = 24, 0 = 67
X2	High blood pressure	Yes(1), No(0)	1 = 30, 0 = 61
X3	Diabetes	Yes(1), No(0)	1 = 5, 0 = 86
X4	Causes of injury	Falling(0), Car Accident(1) Low fall(2), No obvious cause(3)	0 = 50, 1 = 8 2 = 7, 3 = 26
X5	Fracture segment	Lumbar(1), Thoracic(0)	1 = 51, 0 = 40
X6	Previous history	Yes(1), No(0)	1 = 4, 0 = 87
X7	Anti-osteoporosis treatment	Yes(1), No(0)	1 = 33, 0 = 58
X8	Vertebral re-fractures	Yes(1), No(0)	1 = 12, 0 = 79

**Table 3:** Results of multiple linear regression analysis

	Final VAS score			Final ODI score		
	Regression coefficient	Standard error	*P* value	Regression coefficient	Standard error	*P* value
Age (years)	0.003	0.017	0.877	0.022	0.038	< 0.05
Gender	-0.060	0.266	< 0.05	-1.329	0.611	0.133
Height(cm)	-0.014	0.032	0.662	-0.040	0.073	0.587
Weight(kg)	-0.010	0.019	0.605	0.060	0.043	0.169
High blood pressure	-0.254	0.261	0.332	-0.153	0.599	0.799
Diabetes	0.634	0.535	0.240	1.298	1.229	0.295
Causes of injury	0.038	0.095	0.687	-0.545	0.217	< 0.05
Fracture segment	-0.353	0.248	0.159	0.023	0.569	0.968
Length of stay	0.034	0.032	0.285	0.015	0.073	0.839
Previous history	-0.613	0.578	0.292	1.121	1.327	0.401
BMD (T-score)	-0.206	0.242	0.397	-1.425	0.555	0.212
Preoperative VAS score	-0.006	0.114	0.958	-0.037	0.261	0.888
Preoperative ODI score	-0.018	0.029	0.528	0.022	0.067	0.741
Length of surgery(min)	-0.003	0.006	0.539	1.218	0.013	0.995
Bone cement dosage(ml)	0.146	0.074	0.052	0.326	0.170	0.058
Anti-osteoporosis treatment	-0.320	0.257	0.217	-0.849	0.591	< 0.05
Vertebral re-fractures	-0.622	0.400	0.124	0.135	0.919	0.883

*VAS* Visual Analogue Scale, *ODI* Oswestry Disability Index, *BMD* Bone mineral density

## Discussion

Current clinical approaches for the treatment of OVCFs include conservative and surgical treatments. Conservative treatment methods mainly include bed rest, pharmacological analgesia, external fixation with braces, and anti-osteoporosis treatment [8]. However, there are some limitations of conservative treatment, and patients are required to be bed-bound for a long time to brake and be treated symptomatically, which may lead to muscle wasting atrophy in bed-ridden patients and further aggravate the degree of osteoporosis [9], increase the incidence of bed-ridden complications such as venous thrombosis, increase the burden of care, and even patients without strict bed rest will increase the risk of non-union of fractures [10]. Therefore, the current mainstream view is that early minimally invasive surgical intervention for patients with OVCFs is essential to get out of bed and resume daily activities as early as possible [11]. PKP is a relatively mature minimally invasive surgical method [12], and it has become a common procedure for the treatment of OVCFs due to its minimally invasive nature, short operative time, and significant results. By introducing an expandable balloon into the fractured vertebral body, the balloon expands to partially restore the height of the vertebral body [13] and forms a cavity inside the vertebral body, which is filled with bone cement under lower pressure to prevent the vertebral body from continuing to collapse and reinforce and strengthen the vertebral body, achieving a short period of rapid pain relief with minimal surgical trauma and rapid postoperative recovery [14], and having obvious advantages in increasing spinal stability and preventing further compression of the vertebral body. At present, PKP is effective in the treatment of OVCFs in the near term, but there is a lack of large sample size reports on its long-term efficacy in China. The results of this study showed that patients with OVCFs who underwent PKP had better overall results after an average follow-up of more than 10 years, and the VAS scores and ODI scores at the last follow-up were significantly improved compared with those before surgery, suggesting that patients with OVCFs had more satisfactory medium- and long-term outcomes after percutaneous kyphoplasty.

In this study, we found no effect of gender on quality of life ODI scores, but in terms of pain VAS scores, long-term follow-up was less effective in women than in men. It has been suggested that female patients are more sensitive to pain [15], and that sex hormones are associated with gender differences in pain perception. As women age, their estrogen secretion level decreases and they tend to develop osteoporosis [16], which may be the reason why female patients are more likely to experience pain during long-term postoperative follow-up. The effect of age on postoperative outcome is controversial, and the results of this study showed that the older the patient was, the higher the postoperative ODI score was, suggesting that the functional status of her postoperative life was worse, indicating that age is a factor affecting the outcome. OVCFs are insidious and most patients have no obvious trauma or only minor trauma, such as sprains, bumps, slips and falls, or even daily actions such as coughing, sneezing, bending, etc., which can cause vertebral fractures. This is often likened to the "silent disease" [17]. This trend is more pronounced in the postmenopausal female population [18]. Spinal fractures caused by this low-energy injury mechanism are extremely harmful to elderly patients, leading to spinal pain, limitation of motion, and kyphosis [19] resulting in decreased physical function and health-related quality of life, increased complication rates, and mortality [20]. The results of this study showed that the cause of the causative injury was a factor that influenced the ODI score at the final follow-up. The higher the causative violence, the higher the ODI score at the last follow-up and the worse the long-term outcome. This may be due to the greater violence to the spine, greater collapse of the vertebral body, and more damage to the surrounding soft tissues, resulting in slower recovery. Furthermore, individual differences among older adults, such as different degrees of osteoporosis, different sensitivities to pain, and differences in underlying diseases, may affect the daily functional activities of older patients after surgery. These elderly patients all have severe osteoporosis themselves, and there is reactive rapid bone loss after fracture, which is prone to sequential fractures at other sites [21], such as vertebral re-fracture, femoral neck fracture, and carpal fracture, which may have a huge impact on patients' lives, which may have an impact on the outcome of efficacy evaluation.

The treatment of OVCFs is a long-term, complex process. Osteoporosis is one of the irreversible manifestations of the human aging process, and PKP surgical treatment is only one important part of it, and the later anti-osteoporosis treatment is carried out throughout the whole disease [22]. Postoperative standardized anti-osteoporosis treatment was a factor influencing the final follow-up ODI score in the results of this study, and standardized anti-osteoporosis treatment can improve the long-term efficacy of PKP and improve the daily functional activity level of elderly patients. The basic measures of anti-osteoporosis treatment are: adhering to a healthy lifestyle, consuming a balanced diet rich in vitamin D, calcium, low salt and moderate protein, avoiding smoking and alcohol abuse, cautiously using drugs that affect bone metabolism, and performing moderate muscle exercise and rehabilitation therapy. Currently, a popular antiosteoporosis drug regimen is an antiosteoporosis drug plus long-term regular oral calcium and vitamin D [23]. Zoledronic acid is a representative drug of the third generation of bisphosphonates, which has a unique structure containing a double nitrogen imidazole heterocyclic ring, which allows zoledronic acid to bind more strongly to the bone surface [24] and act selectively on bone. One retrospective observational study [25] measured post-treatment changes in bone mineral density and bone turnover markers in 282 postmenopausal women with osteoporosis based on adjustments for treatment duration and zoledronate regimen and found a better prognosis. However, zoledronic acid is relatively expensive to treat, and the impact on health care costs of treating all older women with osteopenia would be substantial. In recent years, a new bone resorption inhibitor, disulfiramab (Proli) an anti-RANKL monoclonal antibody class, has also been used to treat osteoporosis. Disulfiramab is a semiannual subcutaneous injection of 60 mg each with good drug compliance that helps patients improve bone mass and reduce the risk of fractures. Currently, disulfiramab for osteoporosis in postmenopausal women with a high risk of fracture is included in the National Health Insurance Drug List, which greatly reduces the burden on patients. It is important to note that adequate intake of calcium and vitamin D must be taken prior to air therapy to prevent the development of hypocalcemia [26]. Clinical monitoring of calcium levels before each dose and for 2 weeks after the first dose in patients prone to hypocalcemia is recommended. If any patient develops suspected symptoms of hypocalcemia during treatment, blood calcium levels should be measured. In addition, bisphosphonates and disulfiramab are both bone resorption inhibitors and are not recommended for combination use. Some of these patients in our follow-up have also started anti-osteoporosis treatment with disulfiramab in recent years and all reported good results.

In conclusion, the long-term outcome after percutaneous vertebroplasty is more satisfactory, and age, gender, cause of injury, and standardized postoperative anti-osteoporosis treatment may be factors affecting the long-term outcome. There may be some limitations as the follow-up study was retrospective and only some of the patients were followed up. General information about the patients was obtained from the medical records of our hospital. Due to confounding factors, there may be inadequate records, possible selection bias by the researchers in the inclusion of case samples, etc. At the same time, the comparability of data may be affected by the different operating techniques and experience of the performers. The analysis of the long-term efficacy after percutaneous vertebral kyphoplasty and the factors influencing it will be confirmed in the future in a long-term prospective randomized controlled study with a large sample.

## Conclusion

PKP, a current mainstream minimally invasive surgical approach, has proven efficacy in patients with OVCFs. Age, gender, cause of injury, and standardized postoperative anti-osteoporosis treatment may be factors affecting the long-term outcome 10 years after surgery.

### Supplementary Information

**Additional file 1.**

## Data Availability

The datasets used and/or analysed during the current study available from the corresponding author on reasonable request.

## Abbreviations

PKP: Percutaneous kyphoplasty
OVCFs: Osteoporotic vertebral compression fractures
VAS: Visual Analogue Score
ODI: Oswestry Disability Index
BMD: Bone mineral density

## References

[1] Compston JE, McClung MR, Leslie WD (2019). Osteoporosis Lancet.

[2] Ju P, Jiang D (2023). Effects of the obstruction of erector spinae plane in affected people undergoing percutaneous vertebroplasty. BMC Surg.

[3] Wang S, Wang H, Niu L (2018). Clinical efficacy of PVP and PKP in the treatment of OVCFs after bilateral resection of ovarian cancer. Oncol Lett.

[4] Lin F, Zhang Y, Wu T (2022). Local anesthetic and steroid injection to relieve the distal lumbosacral pain in osteoporotic vertebral compression fractures of patients treated with kyphoplasty. Pain Physician.

[5] Wang T, Si F, Zang L (2022). Radiographic adjacent segment degeneration and risk factors for osteoporotic vertebral compression fractures treated with percutaneous kyphoplasty. Int Orthop.

[6] Hou Y, Zhou B, Amuti A (2021). Rapid efficacy of percutaneous kyphoplasty (PKP) in treating thoracolumbar fractures in elderly patients. Am J Transl Res.

[7] Wang W, Liu Y, Wan H (2023). Effectiveness and prognostic factors of different minimally invasive surgeries for vertebral compression fractures. BMC Musculoskelet Disord.

[8] Jeon I, Kim SW, Yu D (2022). Paraspinal muscle fatty degeneration as a predictor of progressive vertebral collapse in osteoporotic vertebral compression fractures. Spine J.

[9] Reginster JY, Beaudart C, Al-Daghri N (2021). Update on the ESCEO recommendation for the conduct of clinical trials for drugs aiming at the treatment of sarcopenia in older adults. Aging Clin Exp Res.

[10] Lee BG, Choi JH, Kim DY (2019). Risk factors for newly developed osteoporotic vertebral compression fractures following treatment for osteoporotic vertebral compression fractures. Spine J.

[11] Yang B, Zhao Y, Zhao Y (2022). Analysis of clinical efficacy after PKP in patients of different genders. Medicine (Baltimore).

[12] Griffoni C, Lukassen JNM, Babbi L (2020). Percutaneous vertebroplasty and balloon kyphoplasty in the treatment of osteoporotic vertebral fractures: a prospective randomized comparison. Eur Spine J.

[13] Hinde K, Maingard J, Hirsch JA (2020). Mortality outcomes of vertebral augmentation (vertebroplasty and/or balloon kyphoplasty) for osteoporotic vertebral compression fractures: a systematic review and meta-analysis. Radiology.

[14] Lin F, Zhang Y, Song X (2023). Percutaneous kyphoplasty to relieve the rib region pain in osteoporotic thoracic vertebral fracture patients without local pain of fractured vertebra. Pain Physician.

[15] Ghazisaeidi S, Muley MM, Salter MW (2023). Neuropathic pain: mechanisms, sex differences, and potential therapies for a global problem. Annu Rev Pharmacol Toxicol.

[16] Geng Q, Gao H, Yang R (2019). Pyrroloquinoline quinone prevents estrogen deficiency-induced osteoporosis by inhibiting oxidative stress and osteocyte senescence. Int J Biol Sci.

[17] Weare-Regales N, Hudey SN, Lockey RF (2021). Practical guidance for prevention and management of glucocorticoid-induced osteoporosis for the allergist/immunologist. J Allergy Clin Immunol Pract.

[18] Chen Z, Lin W, Zhao S (2021). Effect of teriparatide on pain relief, and quality of life in postmenopausal females with osteoporotic vertebral compression fractures, a retrospective cohort study. Ann Palliat Med.

[19] Ren H, Feng T, Hu Y (2022). The value of dynamic fracture mobility in determining the optimum operation choice for acute osteoporotic vertebral compression fracture. J Pain Res.

[20] Jennings JW (2020). Vertebral augmentation is more than just pain palliation, it is about improved mortality. Radiology.

[21] Dai C, Liang G, Zhang Y (2022). Risk factors of vertebral re-fracture after PVP or PKP for osteoporotic vertebral compression fractures, especially in Eastern Asia: a systematic review and meta-analysis. J Orthop Surg Res.

[22] Li W, Wang H, Dong S (2022). Establishment and validation of a nomogram and web calculator for the risk of new vertebral compression fractures and cement leakage after percutaneous vertebroplasty in patients with osteoporotic vertebral compression fractures. Eur Spine J.

[23] Kendler DL, Cosman F, Stad RK (2022). Denosumab in the treatment of osteoporosis: 10 years later: a narrative review. Adv Ther.

[24] Kostyshyn NM, Świetlicka I, Tomaszewska E (2022). Impact of whole body vibration and zoledronic acid on femoral structure after ovariectomy: morphological evaluation. J Clin Med.

[25] Everts-Graber J, Reichenbach S, Gahl B (2022). Effects of zoledronate on bone mineral density and bone turnover after long-term denosumab therapy: observations in a real-world setting. Bone.

[26] Pepe J, Colangelo L, Biamonte F (2020). Diagnosis and management of hypocalcemia. Endocrine.

